# Barriers and opportunities for improving dog bite prevention and dog management practices in northern Indigenous communities

**DOI:** 10.3389/fvets.2023.1199576

**Published:** 2023-09-19

**Authors:** Laurence Daigle, André Ravel, Francis Lévesque, Kabimbetas Noah Mokoush, Yves Rondenay, Audrey Simon, Cécile Aenishaenslin

**Affiliations:** ^1^Département de Pathologie et Microbiologie, Faculté de médecine vétérinaire, Université de Montréal, Saint-Hyacinthe, QC, Canada; ^2^Groupe de Recherche en Épidémiologie des Zoonoses et Santé publique, Faculté de médecine vétérinaire, Université de Montréal, Saint-Hyacinthe, QC, Canada; ^3^Centre de Recherche en Santé Publique de l’Université de Montréal et du Centre Intégré Universitaire de Santé et de Services Sociaux (CIUSSS) du Centre-Sud-de-l’île-de-Montréal, Montreal, QC, Canada; ^4^École d’études autochtones, Université du Québec en Abitibi-Témiscamingue, Val-d’Or, QC, Canada; ^5^Independent Researcher, Kawawachikamach, QC, Canada; ^6^Centre Hospitalier Universitaire Vétérinaire, Faculté de médecine vétérinaire, Université de Montréal, Saint-Hyacinthe, QC, Canada

**Keywords:** dog bites, prevention, epidemiology, Indigenous, northern communities, public health, rabies

## Abstract

Globally, people living in northern Indigenous communities are at higher risk of dog bites than the rest of the population living in North America, with annual incidence ranging from 0.61 to 59.6/10,000 inhabitants. Considering that rabies is endemic in wild canid populations in certain regions of the Arctic, the prevention of dog bites and the management of dog populations are of crucial importance for public health in these contexts. Most northern communities lack access to veterinary services, mainly due to their remote geographical location and to limited financial resources. Currently, northern Indigenous communities are using different approaches and strategies to prevent dog bites and manage dog populations, but the effectiveness of these approaches sometimes lacks evidence, and their low acceptability may affect their implementation. This study aims to describe (1) the current access and uses of veterinary services, and (2) the perceived barriers and opportunities related to dog population management practices currently implemented, or that could be implemented, in a Naskapi community and an Innu community located in northern Quebec (Canada). Quantitative data were collected through a survey to inhabitants on veterinary services (*n* = 122). Qualitative data were collected using individual interviews to inhabitants and health professionals to describe how dog population management measures were perceived, and to identify barriers and opportunities related to their implementation (*n* = 37). Descriptive and inferential analysis (quantitative data) and thematic analysis (qualitative data) were performed. Results show that the two main measures implemented at the time of the study – dog culling and short-duration veterinary clinics – were not perceived as fully acceptable and sustainable. Reinforcing access to veterinary services and other dog-related services, such as shelters and training programs on dogs, was identified as a need to improve dog bites prevention and dog population management in remote Indigenous communities. The implementation of animal health measures should be decided by concerned Indigenous communities to follow decolonial practices. It includes ensuring informed consent of dog owners, improving communication before, during and after interventions, separating veterinary services from rehoming and, most importantly giving back to Indigenous communities the complete leadership over animal health in their communities.

## 1. Introduction

Dog bites have been under the scrutiny of researchers and public health authorities for several years, since they can cause significant impacts on physical and mental health, but also on the economy (1). The severity of bites varies from small lacerations to severe injuries causing death (2). Mental health issues can also be associated with secondary fear, trauma or anxiety related to dog bites (1, 3). In addition, 3–30% of dog bites progress to severe infections, which require medical attention (1, 4–6). The risk of rabies transmission is also a concern in regions where the virus is present in dog or wildlife populations. Costs associated with dog bites include direct costs related to medical care and public health interventions, such as the prevention of rabies. Globally, Hampson and colleagues estimated the annual costs of rabies worldwide at $8.6 billion USD, of which the costs of the administration of rabies post-exposure prophylaxis would account for 20% (7). There are also indirect costs related to medical leave, hospitalization costs, post-traumatic stress treatments and insurance claims (2).

Dog bite incidence varies greatly depending on the local context (8, 9). The incidence at the Canadian level is not well documented. However, based on data of 22 Canadian municipalities between 2003 and 2005, the annual incidence of dog bites is estimated to be 0–9 per 10,000 inhabitants (10). Between 1990 and 2007, 28 fatal cases associated with dog attacks were reported in this country (9). A recent review suggested that people living in northern Indigenous communities are at higher risk of dog bites than the rest of the population, with annual incidence ranging from 0.61 to 59.6/10,000 inhabitants (8). Considering that rabies is endemic in wild canid populations in certain regions of the Arctic (11–13) and that knowledge about dog bites and rabies risks can be limited in some communities (14), the prevention of dog bites and the management of dog populations are of crucial importance in these contexts.

Dog populations in northern Indigenous communities can be challenging to define, since dogs can be kept by individual owners, or they can be considered as community dogs (shared by several owners), feral dogs (no owners), free-roaming owned dogs or owned dogs kept home. Currently, northern Indigenous communities are using different approaches and strategies to prevent dog bites and manage dog populations, including dog population management programs (e.g., spay and neuter clinics), the implementation of laws and regulations to reduce the number of free roaming dogs, dog culling (e.g., most often done using firearms in remote regions due to the lack of other means), as well as training programs to reduce risky behaviors (15–17). However, the effectiveness of these approaches sometimes lacks evidence, and their low acceptability may affect their implementation. In a previous study conducted in one Naskapi community and one Innu community in Canada, perceptions on whether dogs should be tied up or not varied amongst community members (14). Some perceived this measure as necessary, whether others perceived this as a form of abuse or a method that could increase dog aggression. This heterogeneity regarding this particular dog management practice is one example of the importance to better understand the upstream barriers to the implementation of dog management practices.

One major barrier to dog management is that most northern communities lack access to veterinary services, mainly due to their remote geographical location and to limited financial resources (15–18). When those services are available, other types of barriers can affect their use by community members, such as cost, communication issues about services, different perceptions and practices about animal health, and colonial history (15–17, 19, 20). Communities often rely on alternative approaches to manage dog populations, including culling of aggressive, problematic and/or free-roaming dogs in specific situations (e.g., over-abundance). However, if the culling of dogs is sometimes the only available measure to control dog overpopulation, its social acceptability has been described as low in several communities (16, 17).

Sometimes, communities receive short-duration animal health services from different organizations. These services can be administered by veterinary schools (16, 17), by the government or by various non-governmental organizations. For example, in the province of Quebec, the provincial government offers an annual free-of-charge rabies vaccination program for dogs in northern Indigenous communities included in the James Bay and Northern Quebec Agreement (12, 21). The types of veterinary services offered during these short-term services vary, but are usually mostly preventive, including for example vaccination against rabies for dogs, sterilization of dogs and deworming. Some organizations also offer at times other types of interventions, such as dog rehoming, and training programs regarding dog cares. These services vary greatly, lack consistency (17) and little is known about their acceptability and effectiveness (22–24). A better understanding of the contextual elements that impede or facilitate the operationalization of these measures is needed to enhance the effectiveness of dog population management and dog-related risks in northern Indigenous communities.

This study investigates factors influencing the implementation of dog bite prevention and dog population management measures in the context of two northern Indigenous communities located in Quebec, Canada, namely Kawawachikamach (KWW) and Matimekush-Lac John (MLJ). The objectives are (1) to describe the current access and uses of veterinary services, and (2) to identify the perceived barriers and opportunities related to dog population management practices currently implemented or that could be implemented in the future.

## 2. Methods

### 2.1. Author reflexivity statement

Six authors are non-Indigenous people living in southern regions of the province of Quebec. Four of them are veterinarians and researchers, one is a veterinarian-clinician, and one is an anthropologist researcher, all familiar with the context of dogs in northern Indigenous communities. One author is from the Naskapi community living in Kawawachikamach.

### 2.2. Study sites

The study was conducted in two northern Indigenous communities located in Quebec, Canada, namely Kawawachikamach (KWW) and Matimekush-Lac John (MLJ), and one municipality, Schefferville (SCH). KWW is a Naskapi community and MLJ is an Innu community, which are separated by a 15-km road. MLJ is surrounded by the territory of SCH. They are located above the 54th parallel and can only be reached by train or plane. The research team received the approval and support of the Naskapi community of KWW, the community of MLJ and the town of SCH, as part of the larger project into which this study fits called *Balancing Illness and Wellness at the Human-Dog Interface in Northern Canada*.

According to the last census in 2021, the population is 641 inhabitants in KWW (25), 682 in MLJ (26), and 244 in SCH (27). Indigenous people represent 94% of inhabitants in KWW (25), 89% in MLJ (26), and 33% in SCH (27). Naskapi from KWW are beneficiaries of the James Bay and Northern Quebec Agreement, an official status of Quebec residents, created following an agreement between certain Indigenous Nations and governments of Quebec and of Canada (28). James Bay and Northern Quebec Agreement beneficiaries are part of the free-of-charge vaccination program for northern communities. This program aims to offer free rabies vaccination for dogs and to provide technical assistance for the training of local vaccinators, on communities demand (21). In this program, the focus is on rabies vaccination, and there is no surgery for sterilization. In addition, KWW, MLJ and SCH have received short-durations clinics administered by a non-governmental organization. During those visits, many services can be provided, for example, vaccination, sterilization, deworming and sometimes dog rehoming.

### 2.3. Study design

Qualitative and quantitative methods were used for this study. Quantitative data were collected through the administration of a questionnaire to assess the access to and use of veterinary services by community members. Qualitative data were collected through individual interviews to describe how the current dog population management measures were perceived, and to identify barriers and opportunities related to the implementation of future potential measures. Naskapi and Innu local coordinators from KWW and MLJ participated in the recruitment of participants and translation of questions when needed. Data were collected between May 27th and June 12th, 2019. The detailed methodology can be found in Daigle et al. (14), which also presents the main results of a complementary study regarding the knowledge, attitudes and practices toward dogs and dog bites in these communities.

### 2.4. Data collection and analysis

#### 2.4.1. Access to veterinary services

A questionnaire was developed and adapted from tools previously developed by the project team (12). The first part of the questionnaire collected data on KAP on dogs and dog bites [reported in Daigle et al. (14)] (see Supplementary Appendix for the full questionnaire). The study used a convenience sampling strategy targeting adults (over the age of 18 years old; Indigenous or not). Participants were recruited in person in their community with the help of local coordinators, such as in grocery stores, and workplaces. Participants reporting owning at least one dog were asked to answer questions on veterinary services and dog management services available or wished for the community. Descriptive and inferential statistical analyses were carried out using IBM SPSS Statistics (RRID:SCR_016479) version 25 software. Because of the proximity between MLJ and SCH, and the low number of respondents, data from both MLJ and SCH communities were combined (MLJ-SCH) for analysis and compared to data from KWW. Data from MLJ-SCH were compared to data from KWW, meaning that two localities were compared. Statistical tests were performed to test differences in proportions between communities with *p* < 0.05, with either Pearson’s Chi squared test or Fisher Exact test when the theoretical size of any cell was lower than 5. Only significant results are reported. Missing data were excluded to calculate proportions for individual variables.

#### 2.4.2. Barriers and opportunities related to dog population management practices

Semi-structured interviews were conducted with participants representing three categories of community members, in each locality. The three categories include adult (Indigenous or not; over the age of 18 years old) (1) residents who have had a dog bite (or parents of children who have had a dog bite), (2) residents who owned a dog that has bitten a person and (3) health care professionals involved in dog bite management. To recruit community members with these three profiles, we invited participants who completed the questionnaire for the first two categories, and health care professionals were invited to participate in-person when visiting the local health centers in the communities. An interview grid was developed specifically for this project in collaboration with the local coordinators of the involved communities (see Supplementary Appendix for the interview grid). Interviews lasted between 8 and 43 min (mean: 20 min). The first part of the interview collected data on experiences with dog bites, rabies risk perceptions, and the role of health care professionals in dog bites and zoonoses management [reported in Daigle et al. (14)], and the second part explored perceptions of interviewees on how to improve preventive measures and on the main related barriers. All interviews were audio recorded. In order to maintain participants’ confidentiality, localities of the interviewed health care professionals are not mentioned in the results section. Recruitment of interviewees aimed to reach data saturation and capture all opinions on different questions. All interviewees were invited to answer the quantitative survey, if they have not completed it before. The interviews were transcribed and a thematic analysis was performed using NVivo (RRID:SCR_014802) version 12.6 software. Thematic analysis methodology is described in Daigle et al. (14). Briefly, after reading all transcripts, codes were developed according to concepts related to knowledge of dog bites in northern Indigenous communities (8). Transcripts were coded to facilitate the emergence of different themes. A rearrangement of the data was made to analyze the content according to themes.

### 2.5. Ethical committee approval and consent

The interview grid and survey questionnaire were reviewed by one representative of the targeted communities and approved by band councils. Consent forms for both parts were completed or consent was given orally (in English or French) beforehand. Oral consent is an accepted alternative way to obtaining consent in a way to respect the traditional oral information transmission (29). Financial compensation was given to interview participants. Survey respondents were eligible to win a prize (draw). The project protocol was reviewed and approved by the ethical committee at the Université de Montréal (Comité d’éthique de la recherche en sciences et en santé; certificate number #CERSES-19-048-P).

## 3. Results

In total, 122 people completed the survey (Women: 67%; Men: 33%), of which 47 (39%) reported owning at least one dog. Most dog owners (*n* = 38) reported having only one dog. Amongst dog owners, 30% were from KWW and 46% were from MLJ-SCH. In total, 37 inhabitants completed the interview (KWW: 18; MLJ-SCH: 19), of which 66% self-identified as Indigenous.

### 3.1. Veterinary services

#### 3.1.1. Use and awareness of veterinary services in the communities

Services used by dog owners are described in Table 1. Seventy seven percent (77%) declared they have used some veterinary services, the majority (76%) in their communities. Of those who were aware about the availability of some services, vaccination against rabies (32%), sterilization (29%) and vaccination against other diseases (17%) were the most frequently reported. There was no significant difference between communities regarding the use and awareness of veterinary services.

**Table 1:** Reported use and awareness of veterinary services and services that would be desired by dog owners*^†^.

	KWW n (%)	MLJ-SCH n (%)	Total n (%)
*My dog has seen a vet*	9/14 (64%)	24/29 (83%)	33/43 (77%)
Who visited the community	7/9 (78%)	19/25 (76%)	26/34 (76%)
Outside of the community	2/9 (22%)	7/25 (28%)	9/34 (26%)
*Awareness of services available in the community*			
Vaccination against rabies	4/12 (33%)	9/29 (31%)	13/41 (32%)
Vaccination against other diseases	1/12 (8%)	6/29 (21%)	7/41 (17%)
Sterilization	3/12 (25%)	9/29 (31%)	12/41 (29%)
Deworming	2/12 (17%)	5/29 (17%)	7/41 (17%)
Urgent care	0	1/29 (3%)	1/41 (2%)
Euthanasia	0	1/29 (3%)	1/41 (2%)
None	7/12 (58%)	16/29 (55%)	23/41 (56%)
Other	1/12 (8%)	0	1/41 (2%)
*Veterinary services are sufficient*	2/13 (15%)	4/29 (14%)	6/42 (14%)
*It is important that my dog gets vaccinated (rabies and/or core vaccine)*	13/13 (100%)	28/29 (97%)	41/42 (98%)
*Which services would I like to have in my community?*			
Vaccination against rabies	9/13 (69%)	22/27 (82%)	31/40 (78%)
Vaccination against other diseases	12/13 (92%)	19/27 (70%)	31/40 (78%)
Sterilization	10/13 (77%)	18/27 (67%)	28/40 (70%)
Deworming	9/13 (69%)	19/27 (70%)	28/40 (70%)
Urgent care	10/13 (77%)	21/27 (78%)	31/40 (78%)
Euthanasia	6/13 (46%)	12/27 (44%)	18/40 (45%)
None	1/13 (8%)	0	1/40 (3%)

*No significant difference between the two localities (Pearson’s Chi squared test or Fisher Exact).

^†^The number of respondents changes for each question and does not represent the total number of the respondents for each community, because there are missing data. The percentage for each category of each question does not take into account the missing data.

Veterinary services offered in the community were considered insufficient by most respondents (86%) (see Table 1). Access to vaccination against rabies (78%), vaccination against other diseases (78%) and sterilization (70%) were reported as a need in both localities. There was no significant difference between the two localities for the services that would be desired.

Some interviewees perceived that people would be willing to pay for veterinary services. A few interviewees mentioned that the Band council (the local community authority) should pay or should provide help with dog services: “*I think if the community was willing to help with the vet fees, then maybe people would take care of them better, because it would be more of an incentive, right, they would not have to pay out of their hands, they would not have to pay out of their pockets too much money*.” (ID017 – KWW).

#### 3.1.2. Heterogeneous perceptions on short-duration veterinary clinics

Since the interviews took place just after the visit of a non-governmental organization that provided vaccination, sterilization, and rehoming of dogs (named [*charity*] here to preserve anonymity), many interviewees spontaneously shared their perceptions on their services. Reported perceptions of these services were heterogeneous, including positive and negative experiences (Table 2).

**Table 2:** Heterogeneous perceptions of interviewees who spoke about [*charity*] (*n* = 22).

Perceptions	Locality	Interviewees (n)	Health care professional (n)
Positive	KWW	5	1
	MLJ-SCH	7	3
Negative	KWW	3	2
	MLJ-SCH	1	0

Positive aspects reported by the interviewees included a perceived reduction of the dog population size, with positive outcomes for the inhabitants: “*When* [charity] *came, it really lessens the dogs yelping at people at all hours and I think they stopped.*” (ID018 – MLJ-SCH, translation) This reduction was perceived as a relief: “*When they came*, [charity], *they removed 140 strays.* […] *It was stressful all the time with these dogs, all the time in surveillance mode, in attack mode. Because there were males in heat everywhere. Even the children were afraid to play outside. It was dreadful. There, the children are playing outside now.*” (ID003 – MLJ-SCH, translation) Sterilization was perceived as a solution to improve dog behaviors, by one interviewee: “*After the dogs were fixed, the females fixed and the males neutered, there were great results.* […] *And since, after* [the operation of my dog], *he is really calm, gentle, barks less, such a good change for the good. Like the other dogs around that I have seen.*” (ID006 – MLJ-SCH, translation).

However, concerns were reported by interviewees regarding the interventions of the charity. One inhabitant perceived negative impacts on dog behaviors after the clinic: “*I’m disturbed of the situation going on now after the operations and the vaccinations that they had, I find that it is worst that it was before.* […] *Yeah, like their attitude, their aggressiveness is worse than it was before. I mean, we are having more bite issues now than we had before.*” (ID015 – KWW). Another participant mentioned in the interviews that they lost their dog during the visit of the organization: “[…] *they never brought my dog back.* […] *they were only supposed to take dogs that did not have collars and the skinny ones. But they took all the dogs.*” (ID028 – KWW). This interviewee reported that this intervention was conducted without their consent: “*When* [charity] *came here, they took my dog* […] *I did not want them to. She was pregnant and they took her away. They said they would bring her back and they never brought her back and they said they were going to do… I do not know if they did* [the surgery], *they never brought my dog back*.” (ID028 – KWW). Other interviewees were against the concept of taking dogs from northern communities for adoption in “the south.” One health care professional mentioned: “[…] *organizations that pick-up dogs in the North to bring them back to the South, I am completely against that. I find that our animal shelters in the South are already overcrowded. We euthanize all the time, then we take the dogs from the North and bring them back to the South and they are not happier, because they are dogs that are genetically made to be in wide-open spaces.*” (ID009 – Health care professional, translation). One nurse reported being concerned about the possibility of relocating rabid dogs from northern communities to southern locations: “*A few years ago, there was a dog that was sent by* [charity] *or something that had rabies.*” (ID010 – Health care professional, translation).

### 3.2. Barriers and opportunities regarding dog population management practices

#### 3.2.1. Lack of acceptability of dog culling

Although interviewees often recognized that dog culling was the only option available, the lack of acceptability of this practice to control the dog population was frequently reported. Reasons supporting the use of this approach included too many free-roaming dogs or a previous dog bite event: “[…] *there are too many dogs and there is no service here about controlling the dogs or the dog’s population or whatever. That’s why [the local authority] shoot them around here.*” (ID021 – KWW). The lack of acceptability of this practice was shared amongst different types of community members. As one interviewee mentioned: “*But, we do, once a year, bring down the dog population and we get harsh criticism from the* [dispensary] *and from the outside: “What are you guys doing?” But what are you going to do if you got a child that is mauled to death* […].” (ID016 – KWW) One interviewee from the community also perceived this option as unsustainable: “*They do the purge and they think that’s going to fix it, but then, six months later, the same problem comes up.*” (ID017 – KWW).

#### 3.2.2. Need for better dog management services in the communities

Interviewees mentioned improvements that could be implemented to manage dog populations. The availability of a dog shelter was frequently reported as a need in both localities: “*Dogs who are just roaming around all the time could be taken so […] if the owner loses the dog, they would have to go to the rescue shelter and say: “OK, that’s my dog.” And maybe, there, they can be informed.”* (ID025 – KWW) Sixty percent (60%) of the survey respondents were also in favor of a dog shelter (Table 3).

**Table 3:** Reported dog management services that would be desired by dog owners*†.

	KWW n (%)	MLJ-SCH n (%)	Total n (%)
*Which services would I like to have in my community?*			
Dog shelter	5/13 (39%)	19/27 (70%)	24/40 (60%)
Dog obedience training	9/13 (69%)	14/27 (52%)	23/40 (58%)
Dog training program for children/others	9/13 (69%)	15/27 (56%)	24/40 (60%)

*No significant difference between the two localities (Pearson’s Chi squared test or Fisher Exact).

^†^The number of respondents does not represent the total number of the respondents for each community, because there are missing data. The percentage for each category of the question does not take into account the missing data.

Four interviewees mentioned that it would be interesting to have a system to register or identify dogs (without asking this question directly): “*I think part of the solution, chief in Kawawa should hire somebody maybe on a monthly basis, on one month, you work one week as a dog control officer. And you start to identify the dogs with dog tags.”* (ID016 – KWW). Finally, another person suggested that dog care services during the day would help to manage dogs in the community: “[…] *or it could have* […] *like a little dog school, a doggie day care, you know. So, if you do not have time because I work from nine to five, and sometimes, by the time I get home, I’m kind of tired.*” (ID031 – KWW).

#### 3.2.3. Strengthening available training on dogs

Most survey respondents from both localities were also in favor to have dog obedience training for dog owners (58%) and a training program on dog behaviors and care for children or others (60%). The importance of improving access to information and training about dogs and dog management practices was raised by many interviewees: “*I think the more people know, the more they’ll work to be safe, make the dogs safer for the children.* […] *Maybe if the Nation provides training, like how to train your dogs, just to show them what basic needs they need and if there is a financial issue that they’d be willing to help pay, I guess, for a bit of dog food, just maybe a bit of the vaccines because the vaccines and neutering the dogs, it can get pricey for a family*.” (ID017 – KWW).

Many interviewees reported that it would be useful to have prevention promoted in the community either through radio or social media: “[Charity] *used Facebook a lot. Why not. Community radio. People listen to the radio a lot. Facebook and community radio* […]. *Because people, Indigenous people, they are very, very visual*.” (ID006 – MLJ-SCH, translation). However, some interviewees raised concerns about barriers that could be present with training programs. A few people perceived that inhabitants may not prioritize such training: “[people around are not well informed about the risks], *not at all. I do not think they would really care if there was a presentation on that as well* […]. *You see, I was kind of busy, I wasn’t trying to avoid you.*” (ID031 – KWW).

Making training available in schools for kids was suggested as part of the solution: “*Because the kids, mostly will interact with other dogs. They’re the ones outside most of the time.*” (ID025 – KWW) Targeting kids was also considered an interesting option to transfer knowledge to parents: “*And then, kids get home and sometimes even like the kids bring home something from school and tell me something I did not know.*” (ID025 – KWW) Health care professionals from both localities agreed unanimously that children should be taught on dog interactions and how not to trigger aggressive behaviors in dogs. A collaboration between departments of both communities would be interesting, as one nurse mentioned: “*Here, in the schools, it would be very interesting to start working on* [training programs], *in collaboration with the Band Council, the dispensary, the city, the police, to have a good knowledge. People are not well informed about dogs, if there are bites. They know it’s dangerous, but no more than that*.” (ID012 – Health care professional, translation). One interviewee mentioned that the consideration and symbolism of the dog could be taught through the elder’s knowledge transmission: “*I think children should be taught about it. Because before, the elders said that dogs had to be respected, but this teaching seems to be going away. But it should be brought back*.” (ID001 – KWW, translation).

## 4. Discussion

This study describes perceived barriers and opportunities to improve dog bite prevention and dog population management practices in a Naskapi, an Innu, and a settler community located in northern Quebec, Canada. Results show a lack of acceptability of current practices and a need to strengthen access to veterinary and dog-related services. Raising awareness on existing services is also necessary, given that vaccination against rabies was considered unavailable by most dog owners despite the fact that there is a vaccination program offered annually by the government (12, 21).

The two main measures available at the time of the study – dog culling and short-duration veterinary clinics – were not perceived as fully acceptable or sustainable, which is coherent to findings from previous studies conducted in other northern Indigenous communities. In a study conducted in an Inuit village in Nunavik (Quebec, Canada) in 2015, 43% of Inuit participants were against culling biting dogs (12). In another study conducted in Sahtu communities in the Northwest Territories, there were also some ethical concerns and worries about the safety of dog culling when done using firearms (16), a practice that was also criticized by inhabitants of Cree and Assiniboine communities of Saskatchewan (30).

Regarding short-duration veterinary services, they were perceived differently amongst participants in this study. Since permanent veterinary services are not always available in northern communities, it is frequent that remote communities only have access to occasional services (15, 16). Occasional veterinary services have been reported as unsustainable, expensive and sometimes not culturally acceptable in other similar contexts (17, 30). Our study highlighted similar issues and suggests that these types of services need to be improved when offered in these communities. Indeed, the testimony of some interviewees suggests that informed consent of dog owners was not obtained and/or not fully understood prior to some interventions or before the dog was removed from the community for adoption, which raises ethical concerns on how these services are implemented. In Quebec (Canada), it is the responsibility of veterinarians to seek consent from a client before administrating veterinary services to their animal (31). It is also an obligation to provide post intervention services after an intervention (31). All organizations should deploy sufficient efforts and resources to apply these practices when providing veterinary services in these communities. Informed consent from dog owners should also always apply to the rehoming of owned dogs, which constitutes the vast majority of dogs in these communities, even though some of them are free roaming. Historical trauma, structural inequities, and massive culling of dogs during colonization make it essential that Indigenous communities regain control of their own decisions from now on (32, 33). Repatriation of decisions is part of the concept of decolonization (34). Obtaining free and informed consent is absolutely crucial for the implementation of decolonial practices in animal health.

This study also revealed a broader concern regarding rehoming dogs from northern regions, where Arctic fox rabies in endemic, to southern regions where it is absent. The risk of introducing rabid animals in southern regions was raised during the interviews, and it has happened in recent years. Indeed, between 2012 and 2022, five rabid dogs originating from northern Quebec were moved in southern regions for adoption (35). Dissemination of zoonotic pathogens by dog relocation is also noted in other contexts, such as the spread of *Leishmania* spp., rabies, *Brucella* spp., *Echinococcus* spp., or *Leptospira* spp. (36–38). As the rehoming service seems to be the most controversial, an alternative solution would be to separate completely the administration of veterinary services from adoption services in these particular contexts. This could maximize the acceptability of services that have the highest impact on animal and human health, such as rabies vaccination and sterilization of dogs in the community.

Despite these issues in the administration of some services, this study re-iterates the need for better access to veterinary services and other dog-related services such as shelters and training programs in northern Indigenous communities. This need was documented in other communities, including in an Inuit community in Nunavik (Canada) (12). The administration of permanent veterinary services remains a big challenge in northern communities due to the small size of villages, and their remote location. Other studies conducted with northern Indigenous communities have reported similar barriers, including costs related to services (which was also raised in this study), lack of trust of people from outside communities and lack of knowledge and awareness (14, 17, 39). Brook and collaborators (16) suggested that we take examples from the human medical practices in remote areas, where nurses can provide health care, but at distance from medical doctors (16, 40). The use of trained non-veterinarian animal health workers (or para-veterinarian, or also community-based animal health workers (CAHW)) has been implemented in low- and middle-income countries (41). Similar models could be implemented in remote northern communities in order to improve access to veterinary services.

Many interviewees mentioned a desire for training on different subjects on dogs for children and/or for adults, such as dog care, dog management, bite risk avoidance, etc. Gouin and collaborators (42) suggested that a multifaceted educational intervention targeting many stakeholders (children, parents, dog owners and the whole community) could be an avenue to reduce dangerous interactions with dogs leading to dog bites (42). It has been suggested that a multi-pronged approach may be best suited for addressing this issue, as: “*meeting with mushers, dog obedience training classes, outside activities with dogs*” (42). Brook and collaborators (16) combined training of the youth with their implication to the veterinary visits (16). Indeed, they held the clinics in schools. Students were implicated in facilitating communication with locals and training was provided at the same time (16). Some authors have raised uncertainties regarding long-term effects of training programs, since they are not fully evaluated (22–24). More studies are needed to document the long-term effects of training programs for reducing dog bites in the specific context of northern Indigenous communities. It is also important that such programs are developed with and implemented by local people in order to decolonize interventions in Indigenous communities.

Although northern Indigenous communities have their own particularities, it is interesting to note that some of the barriers limiting the implementation of dog population management measures are similar to those observed in other Indigenous communities around the world. For example, facilitating access to veterinary services such as spaying and neutering, as well as building new kennels was also preferred to dog culling by community members in a study conducted in Todos Santos in Guatemala (43). Similarly, lack of access to, high costs and distance of veterinary services were also identified as important barriers to manage dog populations in an aboriginal community of Australia (44). Sharing knowledges and examples of successful, effective and sustainable measures across these communities living with similar dog-related challenges should be highly encouraged in the future.

This study had some limitations. For the recruitment of the survey respondents, inhabitants and health care professionals were recruited through a convenience sampling strategy in order to achieve a certain sample size. Therefore, it is possible that with this strategy, population strata are not all represented adequately according to the source population. Some people that did not answer surveys and did not participate in interviews could have had different opinions on dog management practices. Opinions from interviewees could not represent the entirety of opinions. However, with the strategy of recruiting people until no new opinions are raised, being an iterative process until data saturation is reached, this decreases the risk of missing themes (45). Another limitation is that we only interviewed adults. This could limit our understanding of practices that could be accepted by children. Since this study was exploratory, it focused only on one Innu community and the only Naskapi community worldwide. This may limit the generalizations that can be made from this study and applied to other northern Indigenous communities.

## 5. Conclusion

This study reports on issues about and solutions to improve dog bite prevention and dog population management practices in remote Indigenous communities. Results show a lack of acceptability of some practices, especially regarding dog culling and rehoming of dogs outside the communities. The implementation of animal health measures coherent with a decolonization approach includes ensuring informed consent before the administration of any services, improving communication of actions, providing post-intervention follow-up services, separating veterinary interventions from rehoming services and, most importantly giving back to Indigenous communities the complete leadership over animal health in their communities. It is also essential to evaluate the effectiveness and sustainability of these interventions and measures on dog bites, rabies occurrence and also knowledge, attitudes and practices regarding dog bites and rabies. Indeed, little data exists to date, particularly for remote communities such as northern Indigenous communities.

## Data availability statement

The raw data supporting the conclusions of this article will be made available by the authors, without undue reservation.

## Ethics statement

The interview grid and survey questionnaire were reviewed by one representative of the targeted communities and approved by band councils. Consent forms for both parts were completed or consent was given orally (in English or French) beforehand. Oral consent is an accepted alternative way to obtaining consent in a way to respect the traditional oral information transmission (29) and this method was approved by the Comité d’éthique de la recherche en sciences et en santé (CERSES). Financial compensation was given to interview participants. Survey respondents were eligible to win a prize (draw). The project protocol was reviewed and approved by the ethical committee at the Université de Montréal (Comité d’éthique de la recherche en sciences et en santé; certificate number #CERSES191 19-048-P).

## Author contributions

LD: study design, data collection, analysis and interpretation of data and redaction. AR and CA: study design, contribution to the data analysis and interpretation and revision of the manuscript. FL, KNM, YR, and AS: contribution to result interpretation and final revision of the manuscript. All authors contributed to the article and approved the submitted version.
